# Anomalously Strong Effect of the Ion Sign on the Thermochemistry of Hydrogen Bonded Aqueous Clusters of Identical Chemical Composition

**DOI:** 10.3390/ijms10020507

**Published:** 2009-02-05

**Authors:** Alexey B. Nadykto, Fangqun Yu, Anas Al Natsheh

**Affiliations:** 1 Atmospheric Sciences Research Center, State University of New York at Albany, 251 Fuller Rd., Albany 12203, NY, USA; 2 Kajaani University of Applied Sciences, Kuntokatu 5, 87101 Kajaani, Finland

**Keywords:** Ionic clusters, DFT, *ab initio*, sign preference, Gibbs free energies, hydrogen bonded complexes

## Abstract

The sign preference of hydrogen bonded aqueous ionic clusters *X*±(*H_2_O*)*_i_* (n =1–5, *X* = F; Cl; Br) has been investigated using the Density Functional Theory and *ab initio* MP2 method. The present study indicates the anomalously large difference in formation free energies between cations and anions of identical chemical composition. The effect of vibrational anharmonicity on stepwise Gibbs free energy changes has been investigated, and possible uncertainties associated with the harmonic treatment of vibrational spectra have been discussed.

## 1. Introduction

The importance of a clear and insight understanding of ion-induced nucleation phenomena for a number of issues related to the Earth climate, air quality, public health and various technologies is well established [1–8]. Although the importance of the ion-induced nucleation became recognized long time ago, the pronounced ion sign effect on nucleation rates observed in Wilson’s pioneering experiments [1] in the cloud chamber and known as a sign preference has remained a mystery up until now. Castleman and Tang [6] stated over 30 years ago that “understanding the effect of ions would be very difficult, or even impossible, if the ion’s specific chemical characteristics had a significant effect on their nucleating efficiency”. Recently, Nadykto *et al*. [5] pointed out that the strong effect of ion properties on nucleation rates is essentially quantum in nature, and is controlled by the electronic structure of the core ion through the influence on the intermolecular bonding energies during the initial steps of cluster formation. While core ions considered in [5] differ in both sign and chemical composition, species presented in this study differ in sign only. This allows separating the “sign” and “composition” effects and permits explicit treatment of the “pure” sign preference in aqueous systems. In the present Communication, the thermochemical properties of aqueous clusters *X*^±^(*H_2_O*)*_i_* (n = 1–5, *X* = F; Cl; Br) have been studied using the quantum theory at DFT-PW91PW91/6-311++G(3df,3pd) level. The main goals of the present Communication are to quantify the effect of the ion sign on the thermochemical properties of aqueous clusters of identical chemical composition and to estimate the effect of vibrational anharmonicity on the computed free energies.

## 2. Methods

Initial generated structures were treated initially by semi-empirical PM3 method and then by PW91PW91/6-31+G*. Finally, the most stable (within ∼4 kcal/mole from the lowest energy isomer) structures obtained at PW91PW91/6-31+G* level have been optimized at PW91PW91/6-311++G(3df.3pd) level. PW91PW91/6-311++G(3df,3pd) has been used to obtain both equilibrium geometries and thermochemical properties from computed vibrational spectrums. The PW91PW91 density functional has been used in the combination with the largest Pople basis set 6-311++G(3df,3pd) that provides quite small basis set superposition error (BSSE). In order to ensure the quality of the obtained DFT results, additional MP2/6-311++G(3df,3pd) calculations, both harmonic and anharmonic, have been carried out.

## 3. Results and Discussion

### 3.1. Difference in Formation Free Energies Between Cations and Anions

**Figure 1.**
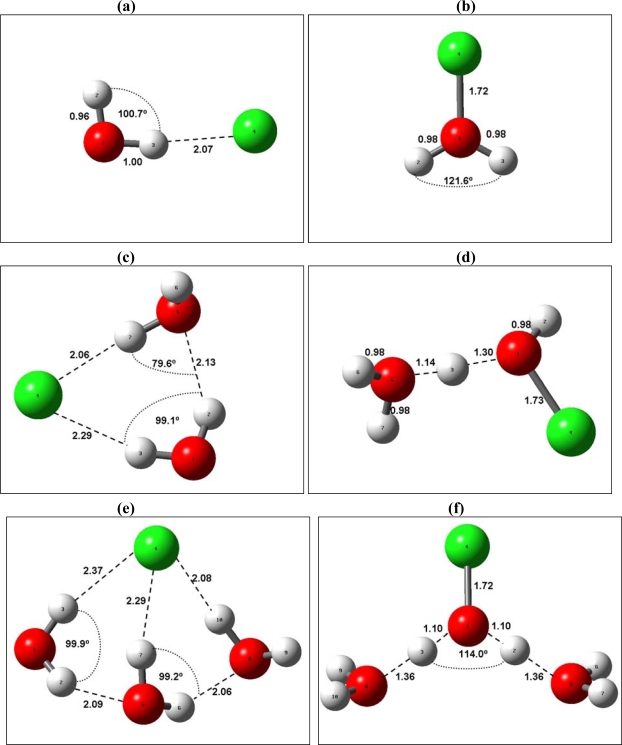

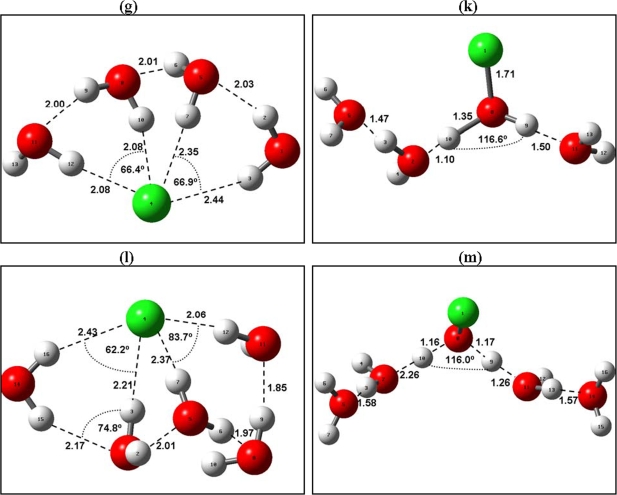
Structures and geometric properties of most stable isomers of *Cl^±^(H_2_O)_i_*, (a) *Cl^−^ (H_2_O)*; (b) *Cl^+^(H_2_O)*; (c) *Cl^−^(H_2_O)_2_*; (d) *Cl^+^(H_2_O)_2_*; (e) *Cl^−^(H_2_O)_3_*; (f) *Cl^+^(H_2_O)_3_*; (g) *Cl^−^(H_2_O)_4_*; (k) *Cl^+^(H_2_O)_4_*; (l) *Cl^−^(H_2_O)_5_*; (m) *Cl^+^(H_2_O)_5_*.

The interest to stepwise Gibbs free energy changes as standalone quantities is related directly to very high sensitivity of nucleation rates to the thermochemistry of initial cluster growth steps. Figure 1 presents the geometries of most stable isomers of *Cl^+^* *(H_2_O)_i_* and *Cl^−^ (H_2_O)_i_*. In addition to most stable isomers, a number of less stable conformers/local minima located within ∼ 5 kcal mol^−1^ of the most stable isomer/global minimum (1–5 for each *n*) have been identified for all the species *F^±^(H_2_O)_i_*, *Cl^±^(H_2_O)_i_*, and *Br^±^(H_2_O)_i_*. Structures of clusters of the same ion sign *F^+^(H_2_O)_i_*, *Cl^+^(H_2_O)_i_* and *Br^+^(H_2_O)_i_*, and *F^−^(H_2_O)_i_*, *Cl^−^(H_2_O)_i_* and *Br^−^(H_2_O)_i_* are similar, while structures of the clusters of the same composition and different sign are quite different. As may be seen from Figure 1, the water molecule in *Cl^−^(H_2_O)_i_* is bonded to the core ion via H-Cl bond, while that in *Cl^+^(H_2_O)_i_* is bonded to the ions via the shorter O-Cl bond. Similar pattern is observed in the case of *F^+^(H_2_O)_i_*, and *F^−^(H_2_O)_i_*, and *Br^+^(H_2_O)_i_* and *Br^−^(H_2_O)_i_*.

**Figure 2.**
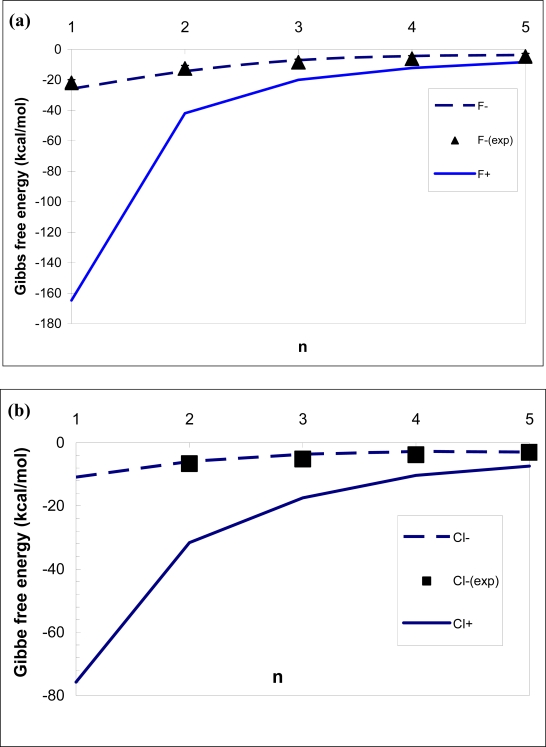

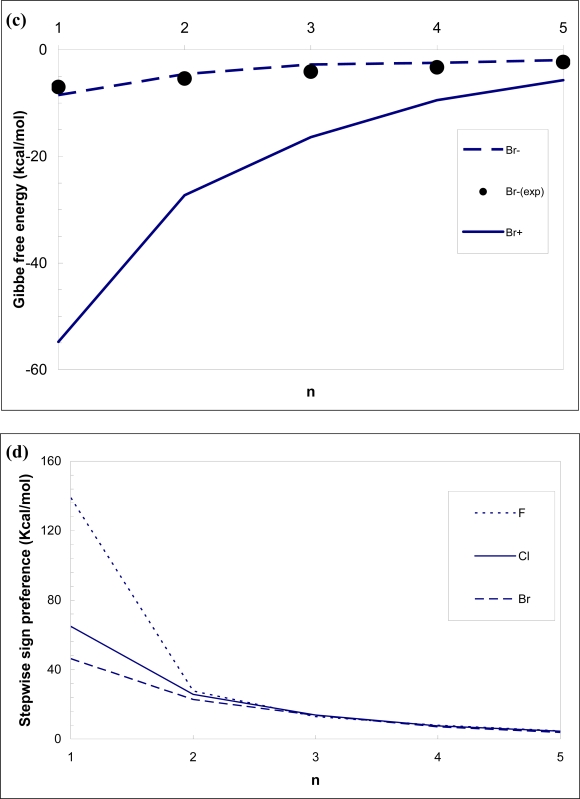
Comparison of experimental and theoretical values of the stepwise Gibbs free energy change Δ*G_n−1,n_* for *X^±^(H_2_O)_i−1_*+ *(H_2_O) ⇔ X^±^(H_2_O)_i_* reactions (a, b, c), and the difference in the Gibbs free energy *δ(j)* between *j*-mers formed over core ions of opposite sign (d). Curves and symbols of refer to theoretical results and experimental data, respectively. Experimental data and theoretical data for *F^−^*(*H_2_O)_n_*, *Cl^−^*(*H_2_O)* and *Br^−^*(*H_2_O)_n_,* were adopted from [11], [5] and [10], respectively. The calculations were performed at the ambient temperature of 298.15 K and ambient pressure of 101.3 KPa.

The difference in the cluster structure has direct impact on the stepwise Gibbs free energy changes associated with the addition of water molecules to the ionic clusters. Figure 2 presents the stepwise Gibbs free energies associated with *X^±^(H_2_O)_i−1_*+ *(H_2_O)* ⇔ *X^±^(H_2_O)_i_* reaction.

As seen from Figure 2, cations have great growth advantage over anions. The difference in the Gibbs free energy is largely associated with the formation enthalpy and it decreases dramatically with the number of molecules in the clusters. The sign preference at the first growth steps is very strong, and the difference in the stepwise Gibbs free energy changes Δ*G_0,1_* for *F^±^(H_2_O)_i_, Cl^±^(H_2_O)_i_* and *Br^±^(H_2_O)_i_* reaches ∼140, 45 and 65 kcal mole^−1^, respectively. This means that cations are much more efficient as nucleators of unary water vapours than anions of identical chemical composition. The stepwise changes in the Gibbs free energies correlate quit well with the mean ion sizes. For example, the hydration of the smallest ion *F^+^(H_2_O)* of 0.149 nm in size (based on the volume calculation) is much stronger than that of bigger *Cl^−^ (H_2_O)* (0.262 nm), *Cl^+^ (H_2_O)* (0.2 nm), *Br^−^(H_2_O)* (0.283 nm), *Br^+^ (H_2_O)*(0.23 nm) and *F^−^(H_2_O)* (0.194 nm).

It is important to note that the sign preference of *Cl^±^(H_2_O)_n_* obtained in molecular-based studies using empirical interaction potential (TI4P) has a different sign. Moreover, the difference between quantum results and Monte-Carlo model predictions using empirical TIP4P water model is excessively large. While the Monte Carlo model [12–13] predicts the thermochemistry of *Cl^−^(H_2_O)_n_* in a good agreement with the present results and experimental data, its deviation in Gibbs free energy for *Cl^+^(H_2_O)_n_* [13] from the present study exceeds several tens of kcal mole^−1^.

### 3.2. Vibrational Anharmonicity

Ongoing discussion [7–8] about the validity of the commonly used harmonic approximation motivated us to perform additional analysis of harmonic and anharmonic cluster spectra and validate them against available experimental data. Tables 1, 2 and 3 present the comparison of the theoretical PW91PW91/6-311++G(3df,3pd) and MP2/6-311++G(3df,3pd) results obtained using the harmonic approximation with anharmonic results and experimental data.

Tables 1, 2 and 3 show clearly that the performance of the harmonic approximation implemented in the framework of the PW91PW91/6-311++G(3df,3pd) method is well beyond satisfactory in all the cases studied here. It is important to note that the application of the anharmonic correction [19] does not lead to any substantial improvement in the theoretical predictions.

**Figure 3.**
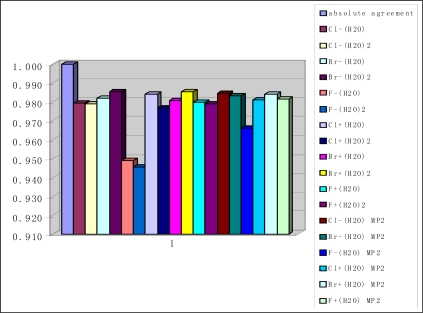
Ratio of anharmonic ZPE to harmonic ZPE.

Another important indication of the reasonable performance of the harmonic approximation implemented in the frame of DFT PW91PW91/6-311++G(3df,3pd) method has been obtained from the comparison of Zero Point Energies (ZPE) computed using the harmonic and anharmonic approximation. As seen from Figure 3 and Table 4, the difference between the harmonic and anharmonic ZPE typically does not exceed 2–3 %. This finding is in excellent agreement with the recent MP2 study [14] of pure water clusters. Another important detail is that DFT results are in good agreement with ab initio MP2 predictions. The contribution of the vibrational anharmonicity to the computed Gibbs free energies does not exceed 0.03–0.2 kcal mol^−1^. This leads us to conclude that anharmonicity is unlikely a source of large uncertainties in the computed Gibbs free energies in all the cases studied here.

## 3. Conclusions

The present study leads us to the following conclusions:

- The effect of ion sign on the formation free energies of aqueous ionic clusters of identical chemical composition is very strong. For example, the difference in the stepwise Gibbs free energy changes Δ*G_0,1_* for *F^±^(H_2_O)_i_*, *Cl^±^(H_2_O)_i_* and *Br^±^(H_2_O)_i_* reaches ∼ 140, 65 and 45 kcal mole^−1^, respectively. It is important to note that the positive sign preference found for unary water vapours does not contradict with the opposite (negative) sign preference observed in recent experiments in binary sulfuric acid-water vapours [20–22]. In contrast to unary clusters, whose stability is controlled by the affinity of water monomers to ions, the stability of more complex binary clusters studied by Froyd and Lovejoy [20,21] and Sorokin *et al*. [22] is controlled by two somewhat competing factors : affinities of H_2_O and H_2_SO_4_ to the ions. While the affinity of water to cations is stronger than that to anions, the affinity of H_2_SO_4_, the key binary nucleation precursor, to ions exhibits the opposite behavior. This leads, due to the very large difference in the affinity of H_2_SO_4_ between anions and cations, to the negative sign preference observed in the experiments [23].
- The harmonic approximation implemented in the framework of the DFT works well in the case of aqueous ionic clusters. Both DFT and ab initio MP2 studies show that the effect of vibrational anharmonicity is mild, and is unlikely a source of large uncertainties in computed free energies.

## Figures and Tables

**Table 1.** Experimental and theoretical frequencies of *F^−^(H_2_O)* (cm^−1^). Subscripts H and A refer to harmonic and anharmonic calculations, respectively.

	F^−^(H_2_O)						F^−^(H_2_O)_2_		
	PW91^H^	PW91^A^	MP2^H^	MP2^A^	Exp^a^	Exp^b^	PW91^H^	PW91^A^	Exp^a^
1	3768	3556	3952	3770	3690	3687	3776	3578	3700
2	1844	1783	2069	953					
3	1623	1609	1715	1625		1650	2717	2375	2520
4	1157	1178	1242	1260		1083–1250	2506	2236	2435
5	569	598	595	586					
6	436	401	412	441					

^a^ [18];

^b^ [17]

**Table 2.** Experimental and theoretical frequencies of *Cl^−^(H_2_O)* (cm^−1^). Subscripts H and A refer to harmonic and anharmonic calculations, respectively.

	Cl^−^(H2O)					Cl^−^(H_2_O)_2_			
	PW91^H^	PW91^H^	MP2^H^	MP2^A^	Exp.	PW91^H^	PW91^A^	exp.1	exp.2
1	3770	3567	3952	3764	3698^a^, 3690^a^, 3699^c^				
2	3069	2740	3376	3161	3285^a^, 3130^a^, 3130^c^	3618	3431	3700^a^	3686^a^
3	1626	1612	1678	1743	1650^b^	3418	3092	3317^a^	3375^a^
4	763	782	794	795	745^c^	3037	2720	3245^a^	3130^a^
5	394	352	387	366					
6	215	204	200	196	210^a^, 155^a^				

^a^ [15] compilation of experimental data;

^b^ [16] compilation of experimental data;

^c^ [17].

**Table 3.** Experimental and theoretical frequencies of *Br^−^(H_2_O)* (cm^−1^). Subscripts H and A refer to harmonic and anharmonic calculations, respectively.

	PW91^H^	PW91^A^	MP2^H^	MP2^A^	Exp^a^
1	3769	3575	3948	3759	3689
2	3223	2871	3506	3257	3270
3	1619	1578	1669	1633	1642
4	668	675	699	690	664
5	323	345	328	310	
6	161	159	158	155	158

^a^ [17]

**Table 4.** Ratio of anharmonic ZPE to harmonic ZPE.

Cl^−^(H_2_O)	0.979	Cl^−^(H_2_O) MP2	0.985
Cl^−^(H2O)_2_	0.979		
Br^−^(H_2_O)	0.982	Br^−^(H_2_O) MP2	0.983
Br^−^(H_2_O)_2_	0.985		
F^−^(H_2_O)	0.949	F^−^(H_2_O) MP2	0.966
F^−^(H_2_O)_2_	0.945		
Cl^+^(H_2_O)	0.984	Cl^+^(H_2_O)MP2	0.981
Cl^+^(H_2_O)_2_	0.977		
Br^+^(H_2_O)	0.981	Br^+^(H_2_O)MP2	0.984
Br^+^(H_2_O)_2_	0.985		
F^+^(H_2_O)	0.980	F^+^(H_2_O) MP2	0.981
F+(H_2_O)_2_	0.979		
